# The effect of peptide adsorption on signal linearity and a simple approach to improve reliability of quantification^☆^

**DOI:** 10.1016/j.jprot.2013.04.034

**Published:** 2013-06-24

**Authors:** Stacey Warwood, Adam Byron, Martin J. Humphries, David Knight

**Affiliations:** a Biological Mass Spectrometry Core Facility, Faculty of Life Sciences, University of Manchester, Manchester M13 9PT, UK; b Wellcome Trust Centre for Cell-Matrix Research, Faculty of Life Sciences, University of Manchester, Manchester M13 9PT, UK

**Keywords:** MRM, multiple reaction monitoring, v/v, volume per volume, Abundance, Linearity, MS, Peptide, Quantification

## Abstract

Peptide quantification using MS often relies on the comparison of peptide signal intensities between different samples, which is based on the assumption that observed signal intensity has a linear relationship to peptide abundance. A typical proteomics experiment is subject to multiple sources of variance, so we focussed here on properties affecting peptide linearity under simple, well-defined conditions. Peptides from a standard protein digest were analysed by multiple reaction monitoring (MRM) MS to determine peptide linearity over a range of concentrations. We show that many peptides do not display a linear relationship between signal intensity and amount under standard conditions. Increasing the organic content of the sample solvent increased peptide linearity by increasing the accuracy and precision of quantification, which suggests that peptide non-linearity is due to concentration-dependent surface adsorption. Using multiple peptides at various dilutions, we show that peptide non-linearity is related to observed retention time and predicted hydrophobicity. Whereas the effect of adsorption on peptide storage has been investigated previously, here we demonstrate the deleterious effect of peptide adsorption on the quantification of fresh samples, highlight aspects of sample preparation that can minimise the effect, and suggest bioinformatic approaches to enhance the selection of peptides for quantification.

**Biological significance:**

Accurate quantification is central to many aspects of science, especially those examining dynamic processes or comparing molecular stoichiometries. In biological research, the quantification of proteins is an important yet challenging objective. Large-scale quantification of proteins using MS often depends on the comparison of peptide intensities with only a single-level calibrant (as in stable isotope labelling and absolute quantification approaches) or no calibrants at all (as in label-free approaches). For these approaches to be reliable, it is essential that the relationship between signal intensity and concentration is linear, without a significant intercept. Here, we show that peptide adsorption can severely affect this relationship, even under controlled conditions, and we demonstrate simple methodologies that can be used to moderate and predict this effect. These findings thus enable the quantification of proteins with increased robustness and reliability.

## Highlights

• First description of effect of peptide adsorption on linearity and quantification
• Demonstration that peptide adsorption affects quantification of fresh samples
• Addition of acetonitrile improves peptide solubility and reliability of quantification

## Graphical abstract

**Figure:**
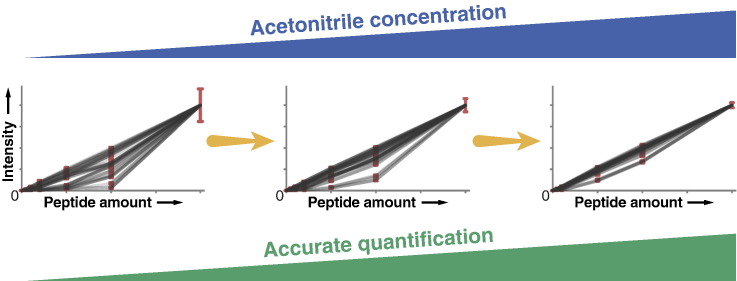

There are many published MS-based approaches for peptide quantification [1–10]. Most of these methodologies rely on the comparison of peptide signal intensities between different samples. Quantification approaches include isotopic labelling, which can be performed at the MS (precursor) or MS/MS level of analysis (e.g. stable isotope labelling by amino acids in cell culture [1] or iTRAQ [2], respectively), allowing samples under comparison to be combined and analysed in a single MS run. Alternatively, label-free quantification involves the comparison of samples analysed in sequential MS runs, which avoids an unfavourable increase in sample complexity [3–5]. Quantification may be targeted, whereby specific target peptides are selected beforehand and quantified, commonly using optimised parameters [6–8]. Targeted quantification may be performed at the MS level of analysis, using extracted ion chromatogram data to monitor the signal of the peptide precursor [9]. Alternatively, some targeted approaches rely on quantification at the MS/MS level of analysis, performed with or without isotopic labelling, such as MRM [10]. For both labelled and label-free approaches, successful peptide quantification requires that the signal intensity observed for a specific peptide or fragment ion has a linear relationship to the abundance of that species in the sample tested. This requirement is of particular relevance to many proteomics methodologies, which do not use calibration curves but instead rely on a single-point calibration. This study tests this critical assumption, demonstrates its impact on the reliability of peptide quantification by MS and provides a simple solution to overcome peptide non-linearity that is compatible with mainstream proteomic approaches.

There are many factors that affect variability in a typical proteomics experiment [11,12]. To reduce the effects of variables that would otherwise confound the interpretation of the relationship between peptide amount and observed signal, we used a low-complexity, commercially available standard in all tests. To assess the linearity of peptide detection, MRM MS was used to quantify a random selection of peptides from a standard six-protein mix tryptic digest (Dionex, Surrey, UK). LC–MS/MS was performed using a NanoAcquity LC (Waters, Manchester, UK) coupled to a 4000 Q TRAP (Applied Biosystems, Framingham, MA, USA). Peptides were injected from polypropylene screw-neck vials (300 μL capacity; Waters), concentrated on a C_18_ trapping column (20 mm length, 180 μm id, 5 μm particle size; Waters) and separated on a bridged ethyl hybrid C_18_ analytical column (75 mm length, 250 μm id, 1.7 μm particle size; Waters) using a 25-min linear gradient from 99% [volume per volume (v/v)] A [0.1% (v/v) formic acid in water]/1% (v/v) B [0.1% (v/v) formic acid in ACN] to 30% (v/v) B at a flow rate of 300 nL/min. Of those peptides that were readily detectable by LC–MS/MS using 75 fmol on column, three peptides were randomly selected for each protein in the six-protein mix. Three MRM transitions per peptide were then selected based on the most intense product ion spectra for each peptide, resulting in a total of 54 transitions (Supporting Information Table S1). The MRM method used a transition dwell time of 50 ms and calculated collision energies (Supporting Information Table S1). The six-protein mix digest was diluted in 0.1% (v/v) formic acid over four orders of magnitude (equivalent to 200 fmol, 100 fmol, 50 fmol, 20 fmol, 10 fmol, 5 fmol, 2 fmol, 1 fmol, 500 amol, 200 amol, 100 amol, 50 amol and 20 amol on column), and each diluted sample was analysed in triplicate by MRM MS. Integrated peak areas were calculated using MultiQuant (version 1.2; Applied Biosystems). Some peptides displayed a linear relationship between signal intensity and peptide amount (Fig. 1). However, many peptides did not display linearity of detection (Fig. 2A); for example, 35% of peptides had an *R*^2^ value of 0.95 or less (Fig. 2A). The non-linearity of detection was consistent between different transitions for the same peptide sequence, indicating that the detector was not saturated (Fig. 1A).

We hypothesised that the apparent non-linear relationship between signal intensity and peptide amount was a result of differential adsorption of analyte to the surface of the polypropylene sample vials or to the sample flow path up to and including the sample loop. This hypothesis is supported by the results of previous studies that described the adsorption of hydrophobic peptide molecules on hydrophobic surfaces in aqueous solution [13], and the effect of peptide adsorption on peptide quantification and recovery [14–16]. Furthermore, the inclusion of an organic modifier has been shown to reduce the loss of specific analytes by surface adsorption [14,17]. However, these studies mainly focussed on losses over time relating to storage stability [15,18] and only tested a limited number of peptides, sometimes as few as one [14–17]. In addition, peptide recovery using a variety of sample vial materials was compared, which demonstrated either little difference between sample vials [15] or improved recovery of peptides using commercially available low-adsorption plastic vials [18]. To our knowledge, the concentration-dependent nature of peptide loss and the direct impact of this on peptide linearity and hence quantification have not been investigated.

To test a simple approach to reduce surface adsorption of peptides and improve peptide quantification, we diluted the six-protein mix digest in 0.1% (v/v) formic acid containing 2.5% (v/v) or 5% (v/v) ACN. We used ACN because this organic modifier is routinely used for LC separations upstream of MS analysis, and it results in reduced ion suppression compared to aprotic solvents such as DMSO. The maximum concentration of ACN tested was 5% (v/v) in order to reduce loss of hydrophilic peptides during LC separation. All samples were analysed identically and in triplicate. The addition of 2.5% (v/v) ACN increased the observed linearity of detection of all peptides, which increased further in the presence of 5% (v/v) ACN (Fig. 1D–F; Supporting Information Fig. S1; Supporting Information Fig. S2). The proportion of peptides with an *R*^2^ value of 0.95 or less was reduced from 35% in formic acid alone to 0% in the presence of 5% (v/v) ACN, whereas the proportion of peptides with an *R*^2^ value of greater than 0.99 was increased from 35% in formic acid alone to 83% in the presence of 5% (v/v) ACN (Fig. 2A). The addition of 5% (v/v) ACN was sufficient to correct the linearity of detection of the majority of peptides tested, although the most hydrophobic of peptides may require higher concentrations of ACN. These data indicate that, in the presence of organic solvent, the peptide mixtures were more accurately quantified. Furthermore, the proportion of measurements with an RSD of 5% or less was 41% in formic acid alone, compared to 67% in the presence of 5% (v/v) ACN (Fig. 2B). This indicates that the presence of organic modifier increased the precision of measurements by MS.

Peptide hydrophobicity has been shown to influence the detectability of peptides using MS [19–22], but its effect on accurate quantification is not clear. To examine the relationship between peptide hydrophobicity and non-linearity of detection, we calculated a number of parameters of peptide properties. The LC retention time of each peptide was recorded as an indicator of hydrophobicity; peptides with longer retention times were considered to be more hydrophobic [23]. In addition, the GRAVY score [24] of each peptide was calculated using the ProtParam tool (http://expasy.org/tools/protparam.html) on the expert protein analysis system proteomics server from the Swiss Institute of Bioinformatics [25]. Notably, the more hydrophobic peptides (with longer retention times and higher GRAVY scores) generally had lower linearity of detection as determined by lower *R*^2^ values (Fig. 2C, D). Moreover, the linearity of detection of all peptides was increased in the presence of organic solvent, including the most hydrophobic peptides. Six out of seven peptides with a GRAVY score less than 0.1 had an *R*^2^ value of greater than 0.99 in the presence of 2.5% (v/v) ACN, whereas peptides with a GRAVY score greater than 0.1 generally had a lower *R*^2^ value (Fig. 2D). In the presence of 5% (v/v) ACN, 15 out of 18 peptides had an *R*^2^ value of greater than 0.99 (Fig. 2C, D). Our data suggest that more hydrophilic peptides should be selected for MS studies in order to provide more reliable quantification. Of the peptides tested here, the addition of 2.5% (v/v) ACN was sufficient to allow reliable quantification of peptides with a GRAVY score of less than 0.1. Increasing the concentration of ACN to 5% (v/v) allowed the majority of peptides to be reliably quantified. These data show that observed peptide retention time and predicted peptide hydrophobicity provide an indication of reliability for linearity of detection, which may direct the selection of peptides for reliable quantification by MS.

Together, these data suggest that peptide solubility and its concentration-dependent role in surface adsorption affect the relationship between peptide amount and signal intensity. The work presented here examines the fundamentals of this effect in a simple, well-defined system, but it is also relevant for more complicated mixtures, in which the potential for competitive adsorption would increase the complexity of the effect. Non-linearity of response presents a significant hurdle to quantitative protein MS, particularly because it cannot be corrected by the presence of an internal standard. The addition of organic modifier increased the linearity and sensitivity of peptide detection, probably due to reduced surface adsorption, which increased both the accuracy and precision of quantification by MS. Computational tools have been developed to predict readily detectable peptides as surrogates for protein quantification [19–22], but such tools do not assess the reliability of peptide quantification. We propose that the evaluation of peptide hydrophobicity could be used as a predictor of the linearity of peptide detection and thus provide a useful selection criterion for peptides most likely to be reliably quantified in targeted MS experiments. Furthermore, we present a general method for reducing non-linearity of peptide detection, which provides a straightforward solution to improve the reliability of peptide quantification in MS-based quantification studies.

## Conflict of interest

The authors declare no conflict of interest.

## Figures and Tables

**Fig. 1:**
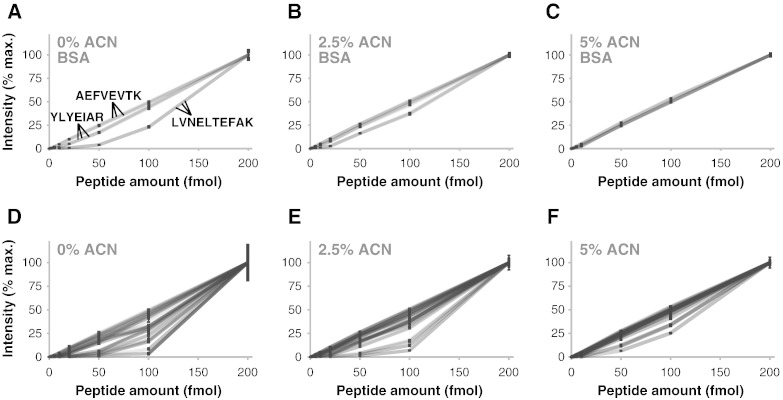
Relationship between peptide amount on column and signal intensity measured by MRM MS. (A–C) Relative signal intensity is displayed for the three transitions measured for each of three BSA peptides in 0.1% (v/v) formic acid (A), 2.5% (v/v) ACN (B) or 5% (v/v) ACN (C). Peptide sequences are indicated. (D–F) Relative signal intensity is displayed for all 54 transitions measured for all selected peptides in 0.1% (v/v) formic acid (D), 2.5% (v/v) ACN (E) or 5% (v/v) ACN (F). All measurements were acquired in triplicate; mean points are plotted, and error bars represent SD. Lines are shaded transparent grey to visualise overlapping lines.

**Fig. 2:**
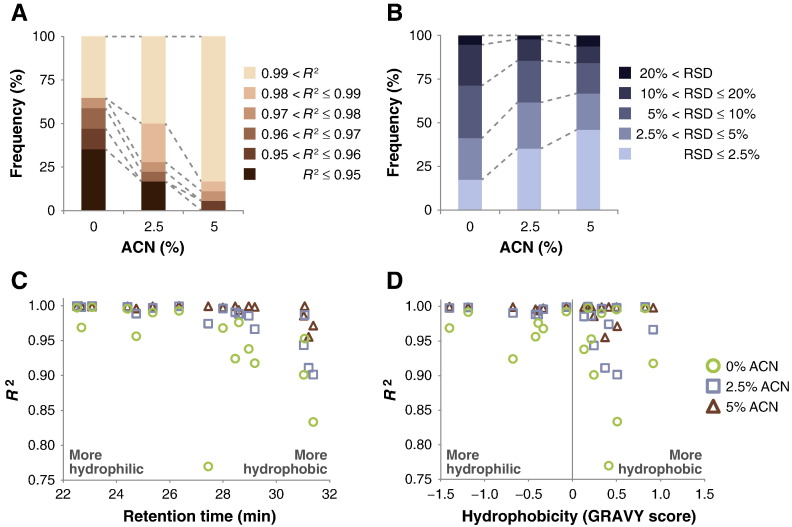
Effect of organic modifier on accuracy and precision of peptide quantification by MS. (A) Distribution of *R*^2^ values of all peptides in the presence or absence of ACN. *R*^2^ values were calculated from the signal intensities of each transition as a function of peptide amount on column using the least-squared method. Mean *R*^2^ values were generated for each peptide. (B) Distribution of RSD values of all transitions in the presence or absence of ACN. RSD values were calculated from the signal intensities for each transition. (C and D) Mean *R*^2^ values of all peptides in the presence or absence of ACN were compared to the hydrophobicity of each peptide as determined by the LC retention time (C) or the GRAVY score (D). More hydrophobic peptides have a longer retention time and a higher GRAVY score; more hydrophilic peptides have a shorter retention time and a lower GRAVY score.

## Appendix A Supplementary data

Supplementary material. Table S1 – MRM transitions and parameters used for MS analyses. Fig. S1 – The relationship between peptide amount on column and signal intensity by MRM MS for all peptides tested in the presence or absence of ACN. Fig. S2 – The relationship between peptide amount on column and signal intensity by MRM MS for all peptides tested in the presence or absence of ACN (log-log plot).
