# Supplementary material for: The effect of peptide adsorption on signal linearity and a simple approach to improve reliability of quantification
Source: J Proteomics. 2013 Jun 24;85:160–4. doi: 10.1016/j.jprot.2013.04.034 (PMC3694305; doi:10.1016/j.jprot.2013.04.034)
Supplement: Supplementary material. — Table S1 – MRM transitions and parameters used for MS analyses. Fig. S1 – The relationship between peptide amount on column and signal intensity by MRM MS for all peptides tested in the presence or absence of ACN. Fig. S2 – The relationship between peptide amount on column and signal intensity by MRM MS for all peptides tested in the presence or absence of ACN (log-log plot). [file mmc1.pdf]

SUPPORTING INFORMATION

The effect of peptide adsorption on signal linearity and a simple approach to improve reliability of quantification

Stacey Warwood[1], Adam Byron[1], Martin J. Humphries, David Knight*

[1] These authors contributed equally to this work.

*Corresponding author:

Dr David Knight, Biological Mass Spectrometry Core Facility, Faculty of Life Sciences, University of Manchester, Manchester M13 9PT, UK.

Tel.: +44 (0) 161 2751561.

Fax: +44 (0) 161 2755082.

E-mail: david.knight@manchester.ac.uk

Table S1 – MRM transitions and parameters used for MS analyses. Peptides selected for analysis from the six-protein mix digest are listed alongside the transitions. Precursor ions selected in the first quadrupole (Q1) and product ions monitored in the third quadrupole (Q3) are reported as *m*/*z* values.

Protein	Peptide	Transitions (*m*/*z*) (Q1 / Q3a, Q3b, Q3c)	Collision energies (eV) (Q3a, Q3b, Q3c)	Declustering potentials (DP) (Q3a, Q3b, Q3c)
Serotransferrin	LCQLCAGK	476.2 / 549.3, 677.3, 838.3	24.3, 24.3, 24.3	75, 75, 75
	ILESGPFVSCVK	668.8 / 1110.5, 894.4, 981.5	28.9, 33.9, 33.9	75, 75, 75
	CGLVPVLAENYK	682.4 / 933.5, 624.3, 737.4	29.6, 44.6, 39.6	75, 75, 75
Bovine serum albumin	AEFVEVTK	461.8 / 476.3, 575.3, 722.4	28.6, 23.6, 18.6	55, 55, 75
	YLYEIAR	464.3 / 488.3, 651.3, 764.4	28.7, 23.7, 23.7	75, 75, 75
	LVNELTEFAK	582.3 / 595.3, 708.4, 951.5	29.6, 29.6, 29.6	75, 75, 75
Alcohol dehydrogenase	ANELLINVK	507.3 / 586.4, 699.5, 828.5	25.9, 20.9, 20.9	55, 55, 75
	EKDIVGAVLK	536.3 / 586.4, 699.5, 814.5	32.3, 32.3, 27.3	75, 75, 110
	VVGLSTLPEIYEK	724.4 / 1079.6, 1249.7, 778.4	31.7, 31.7, 31.7	75, 75, 75
Lysozyme	GTDVQAWIR	523.3 / 545.3, 673.4, 772.4	26.7, 26.7, 26.7	75, 55, 75
	FESNFNTQATNR	714.8 / 1152.5, 804.4, 951.5	33.7, 33.7, 33.7	75, 110, 75
	GYSLGNWVCAAK	663.8 / 1019.5, 1106.5, 906.4	36.2, 36.2, 36.2	75, 55, 75
Cytochrome c	EDLIAYLK	482.8 / 494.3, 607.4, 720.5	19.6, 24.6, 24.6	75, 55, 55
	TGPNLHGLFGR	584.8 / 549.3, 686.4, 799.5	39.7, 34.7, 39.7	110, 110, 110
	KTGQAPGFSYTDANK	528.9 / 548.3, 711.3, 798.4	24.2, 24.2, 19.2	75, 55, 55
β-Galactosidase	GDFQFNISR	542.3 / 489.3, 636.3, 764.4	27.6, 27.6, 27.6	75, 75, 75
	LTAACFDR	477.7 / 669.3, 740.3, 841.4	24.4, 24.4, 24.4	55, 55, 75
	APLDNDIGVSEATR	729.4 / 1061.5, 1176.5, 832.5	42.0, 42.0, 42.0	75, 75, 110

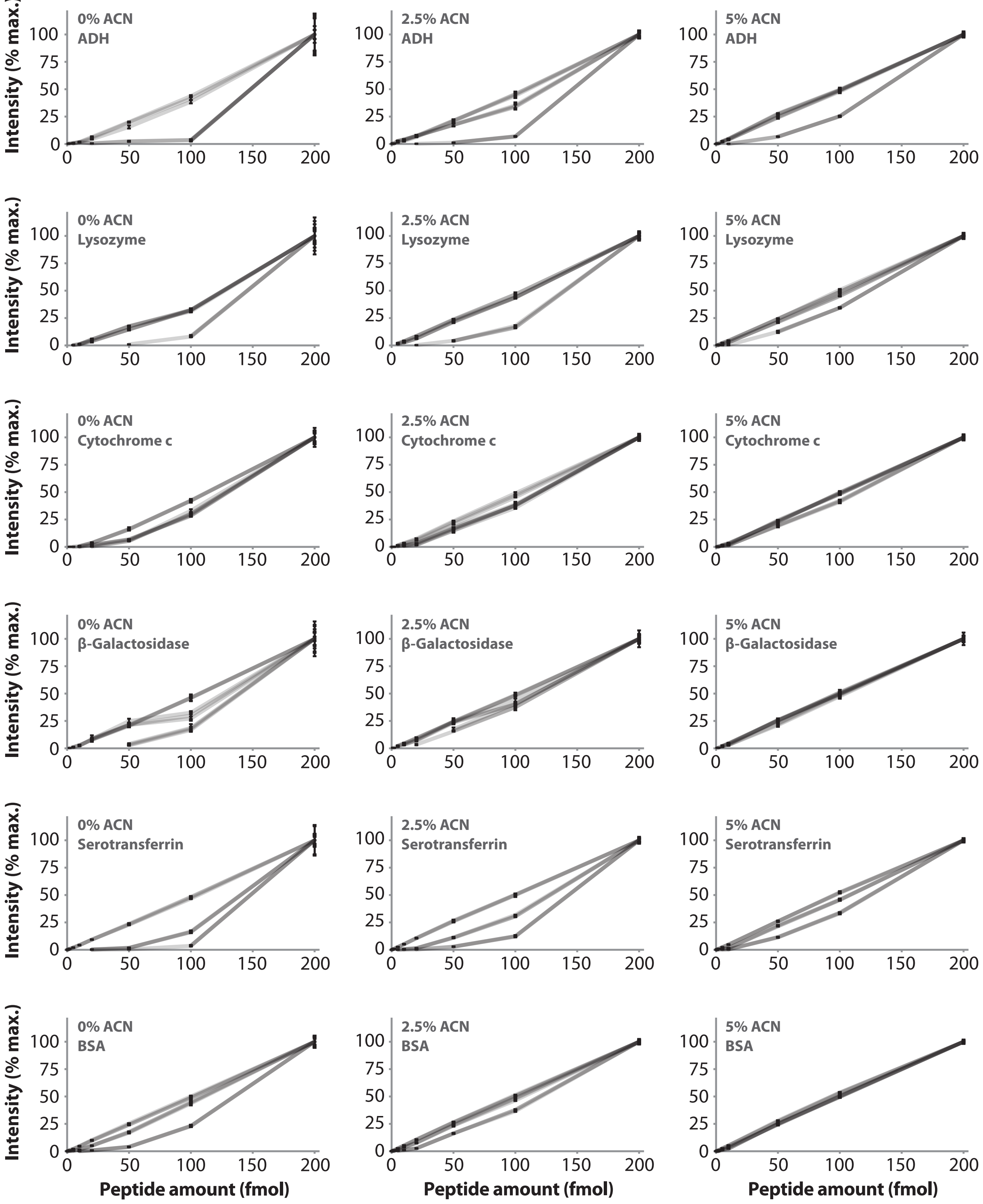

Fig. S1 – The relationship between peptide amount on column and signal intensity by MRM MS for all peptides tested in the presence or absence of ACN. All measurements were acquired in triplicate and are displayed as mean ± SD. Lines are shaded transparent grey to visualise overlapping lines. Abbreviations: ACN, acetonitrile; ADH, alcohol dehydrogenase; BSA, bovine serum albumin.

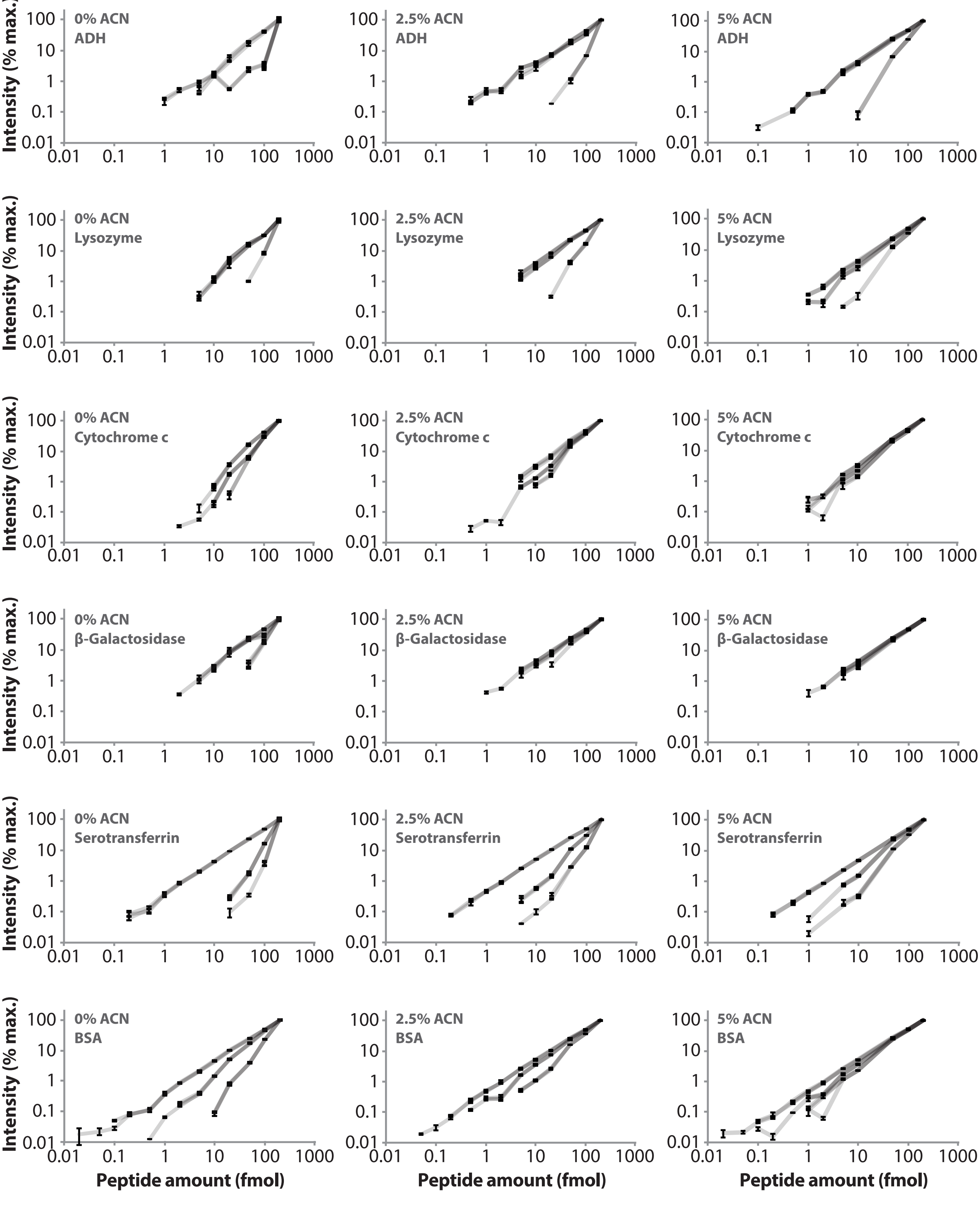

Fig. S2 – The relationship between peptide amount on column and signal intensity by MRM MS for all peptides tested in the presence or absence of ACN (log-log plot). All measurements were acquired in triplicate and are displayed as mean ± SD. Lines are shaded transparent grey to visualise overlapping lines. Abbreviations: ACN, acetonitrile; ADH, alcohol dehydrogenase; BSA, bovine serum albumin.
